# Spatial characterization and quantification of CD40 expression across cancer types

**DOI:** 10.1186/s12885-023-10650-7

**Published:** 2023-03-09

**Authors:** Katherine M. Bates, Ioannis Vathiotis, Tyler MacNeil, Fahad Shabbir Ahmed, Thazin Nwe Aung, Yuliya Katlinskaya, Sabyasachi Bhattacharya, Amanda Psyrri, Steven Yea, Amanda Parkes, Nooshin Hashemi Sadraei, Siddhartha Roychoudhury, David L. Rimm, Niki Gavrielatou

**Affiliations:** 1 Department of Pathology, Yale School of Medicine, 310 Cedar Street, BML 112, New Haven, CT 06510-8023 USA; 2 Department of Pathology, Wayne State University, Detroit, MI USA; 3 Amgen, Thousand Oaks, CA USA; 4 Department of Internal Medicine, Section of Medical Oncology, Attikon University Hospital, Athens, Greece

**Keywords:** CD40, Quantitative immunofluorescence, Cancer cells, NSCLC, Ovarian, Pancreatic

## Abstract

**Background:**

CD40, a TNF receptor family member, is expressed by a variety of immune cells and is involved in the activation of both adaptive and innate immune responses. Here, we used quantitative immunofluorescence (QIF) to evaluate CD40 expression on the tumor epithelium of solid tumors in large patient cohorts of lung, ovarian, and pancreatic cancers.

**Methods:**

Tissue samples from nine different solid tumors (bladder, breast, colon, gastric, head and neck, non-small cell lung cancer (NSCLC), ovarian, pancreatic and renal cell carcinoma), constructed in tissue microarray format, were initially assessed for CD40 expression by QIF. CD40 expression was then evaluated on the large available patient cohorts for three of the tumor types demonstrating high CD40 positivity rate; NSCLC, ovarian and pancreatic cancer. The prognostic impact of CD40 expression on tumor cells was also investigated.

**Results:**

CD40 expression on tumor cells was found to be common, with 80% of the NSCLC population, 40% of the ovarian cancer population, and 68% of the pancreatic adenocarcinoma population displaying some degree of CD40 expression on cancer cells. All of three of these cancer types displayed considerable intra-tumoral heterogeneity of CD40 expression, as well as partial correlation between expression of CD40 on tumor cells and on surrounding stromal cells. CD40 was not found to be prognostic for overall survival in NSCLC, ovarian cancer, or pancreatic adenocarcinoma.

**Conclusions:**

The high percentage of tumor cells expressing CD40 in each of these solid tumors should be considered in the development of therapeutic agents designed to target CD40.

**Supplementary Information:**

The online version contains supplementary material available at 10.1186/s12885-023-10650-7.

## Introduction

Cluster of differentiation 40 (CD40) is a transmembrane costimulatory protein and member of the tumor necrosis factor receptor family. As one half of the CD40/CD40L axis, CD40 is often described as bridging the innate and adaptive immunity [1, 2]. CD40 is expressed on antigen-presenting cells (APCs), such as dendritic cells, myeloid cells, and B cells, and interacts with CD40 ligand (CD40L) on activated CD4 T cells, B cells, and natural killer (NK) cells [1, 3]. As part of the adaptive immune response, the CD40/CD40L axis mediates antigen-specific T and B cell activation and is therefore involved in both cell-mediated and humoral immunity [1, 4]. The CD40/CD40L interaction also plays a role in the innate immune response by activating NK cells [1, 5].

Due to the importance of the CD40/CD40L axis in antigen-specific immune responses especially, cancer therapeutics involving CD40 have been and continue to be explored. The goal of these CD40 targeted therapies, which include agonistic CD40 antibodies, bispecific antibodies with CD40 as one of the targets, and adenoviral vectors that increase CD40L expression, is to evoke a primarily tumor-specific T cell response [1, 2, 6, 7]. In addition, CD40 agonists have been shown to increase tumor immunogenicity, and thus, act synergistically to immune checkpoint blockade [8]. Despite the well-established rationale for their use, many CD40 therapies never reach testing in clinical trials due to dose-limiting toxicity, which oftentimes leads to suboptimal efficacy [6]. Notably, in clinical trials, several of the CD40 targeted therapies have been linked to high risk of cytokine release syndrome, thrombocytopenia, and hepatotoxicity [6].

Another important consideration for the use of CD40-targeting agents, is the specific immune-, or other, cell-phenotypes whose functions are modulated. While these therapies aim to target CD40 molecules expressed on APCs, previous studies have reported the presence of CD40 on a variety of other cell types, including macrophages, monocytes, platelets, fibroblasts, epithelial cells, endothelial cells, and most importantly tumor cells [1, 3, 9–11]. It has been proposed that the adverse events specifically seen with CD40 agonists are a result of widespread CD40 expression by hematopoietic cells [6]. However, it may be worth further examining CD40 expression by tumor cells, as it could affect the efficacy of drugs targeting CD40.

Due to the use of CD40 as a target of therapeutics, a comprehensive and quantitative assessment of CD40 expression on the tumor epithelium may help guide drug development and/or the patient selection criteria. Here, we employed quantitative immunofluorescence (QIF) to assess the expression levels of CD40 in several tumor types. CD40 distribution and patterns of expression were further explored in larger cohorts of non-small cell lung cancer (NSCLC), ovarian cancer, and pancreatic adenocarcinoma, and correlated with clinicopathologic characteristics and outcome.

## Methods

### Patient cohorts and TMA construction

All assays were performed on formalin-fixed paraffin-embedded patient-tissue specimens from Yale New Haven Hospital, which were collected from surgical resections or biopsies, and then constructed into tissue microarray (TMA) format. TMAs from nine different solid tumors (bladder, breast, colon, gastric, head and neck, NSCLC, ovarian, pancreatic, and renal cell carcinoma) were used for the evaluation of CD40 expression across cancer types (Supplementary Table 1). Population-based assessment of CD40 expression, correlation with clinicopathologic features, and prognostic significance was completed using retrospectively collected cohorts of NSCLC (YTMA423), ovarian cancer (YTMA264), and pancreatic adenocarcinoma (YTMA454), which were selected for assessment due to tissue availability. Clinical annotations for these cohorts were retrieved from clinical records and pathology reports, and the baseline characteristics of these cohorts can be found in Table 1. Treatment data were not available for these cohorts, so this study does not examine the predictive value of CD40 expression for specific treatments. The need for Informed Consent was waived by the Yale Human Investigation Committee (protocol #9,505,008,219) due to the retrospective nature of the study. The study was approved by the Yale Human Investigation Committee protocol #9,505,008,219 and conducted in accordance with the Declaration of Helsinki.

**Table 1:** Correlation of clinicopathological characteristics with and prognostic assessment of CD40 expression, divided into low CD40 expression and high CD40 expression categories by median. (OS, overall survival)

Characteristic	NSCLC cohort (YTMA423), N (%)			Ovarian cancer cohort (YTMA264), N (%)			Pancreatic cancer cohort (YTMA454), N (%)		
	CD40 low *N* = 107	CD40 high *N* = 107	*p*-value	CD40 low *N* = 50	CD40 high *N* = 50	*p*-value	CD40 low *N* = 69	CD40 high *N* = 68	*p*-value
**Age**	67 [43, 89]	69 [43, 84]	0.17	62 [17, 85]	65 [34, 91]	0.41	71 [46, 87]	70 [34, 86]	0.6
**Gender**			0.89			n/a			0.31
Female	67 (63%)	65 (61%)		50 (100%)	50 (100%)		33 (48%)	39 (57%)	
Male	40 (37%)	42 (39%)		n/a	n/a		36 (52%)	29 (43%)	
**Race**			> 0.99			0.24			0.76
White	99 (93%)	98 (92%)		50 (100%)	47 (94%)		64 (93%)	62 (91%)	
Non-white	8 (7%)	9 (8%)		0 (0%)	3 (6%)		5 (7%)	6 (9%)	
**Smoking (current/former)**			0.16			n/a			> 0.99
Yes	97 (91%)	89 (83%)		n/a	n/a		40 (58%)	42 (62%)	
No	10 (9%)	18 (17%)		n/a	n/a		26 (38%)	26 (38%)	
Unknown	n/a	n/a					3 (4%)	0 (0%)	
**Stage**			0.96			0.48			0.39
I	73 (68%)	74 (69%)		13 (26%)	10 (20%)		5 (7%)	1 (1%)	
II	26 (24%)	26 (24%)		3 (6%)	6 (12%)		62 (90%)	64 (94%)	
III	8 (7%)	7 (7%)		33 (66%)	31 (62%)		1 (1%)	2 (3%)	
IV	n/a	n/a		1 (2%)	3 (6%)		1 (1%)	1 (1%)	
**Histology**			0.18			0.16			> 0.99
Squamous (NSCLC only)	24 (22%)	33 (31%)		n/a	n/a		n/a	n/a	
Adenocarcinoma	80 (75%)	68 (64%)		9 (18%)	10 (20%)		68 (99%)	67 (99%)	
Serous (ovarian cancer only)	n/a	n/a		18 (36%)	26 (52%)		n/a	n/a	
Other	3 (3%)	6 (6%)		23 (46%)	14 (28%)		1 (1%)	1 (1%)	
**OS (months)**	49 [0.3, 96]	50 [0.2, 95]	0.27	27 [3, 153]	42 [2, 346]	0.25	22 [4, 95]	37 [1.3, 107]	0.47

### Quantitative IF

TMA slides were deparaffinized, rehydrated with an ethanol gradient, and subjected to heat-mediated antigen retrieval in an ethylenediaminetetraacetic acid buffer of pH 8, for 20 min at 97 °C in a pressure heating container (PT module, Lab Vision, Fremont, CA, USA). Next, endogenous peroxidases were inactivated with 2.5% hydrogen peroxide in methanol for 30 min, followed by a 30 min incubation with 0.3% bovine serum albumin with 0.05% Tween-20 blocking solution. The slides were then incubated with primary antibodies to detect CD40 (Clone EPR20540, Abcam, Waltham, MA, USA) and cytokeratin (Clone AE1/AE3, Agilent, Santa Clara, CA, USA) at 4 °C overnight. An isotype-specific horseradish peroxidase-conjugated secondary antibody and tyramide-based amplification system was used for signal detection of the target, while an isotype-specific secondary antibody conjugated to a fluorophore was used for signal detection of cytokeratin. Finally, all nuclei were stained with 4’,6-diamino-2-phenylindole (DAPI).

Fluorescent images were obtained using a PM-2000 scanning platform (Navigate Biopharma, Carlsbad, CA, USA), and signal quantification was performed with the automated quantitative analysis (AQUA™) method of quantitative immunofluorescence (QIF). For our analysis, the target (CD40) was measured in 3 molecular compartments: (1) the tumor mask, which was created by binarizing and dilating the cytokeratin signal, (2) the whole histospot mask, which was created by binarizing and dilating the DAPI signal, and (3) the stroma mask, which was created by subtracting the tumor mask from the whole histospot mask, representing all non-malignant cells within the tumor microenvironment. QIF scores were generated by dividing the summed pixel intensities of the target by the area of the compartment in which it was measured, and then normalizing to the exposure time and bit depth at which the images were captured. Spots were designated as positive or negative for CD40 expression by the visual cutpoint, defined as the lowest QIF score with a clear membranous or cytoplasmic localization of the signal, and a signal that is distinguishable from background noise. Representative images for each tissue type were inspected by a board-certified pathologist, and spots with staining artifacts or insufficient (< 2%) compartment area were excluded from analysis after visual inspection. For the NSCLC, ovarian cancer, and pancreatic adenocarcinoma cohorts, two TMA blocks were analyzed, each containing one tumor core per patient, and the QIF scores were averaged for each case.

### Antibody validation

**Fig. 1:**
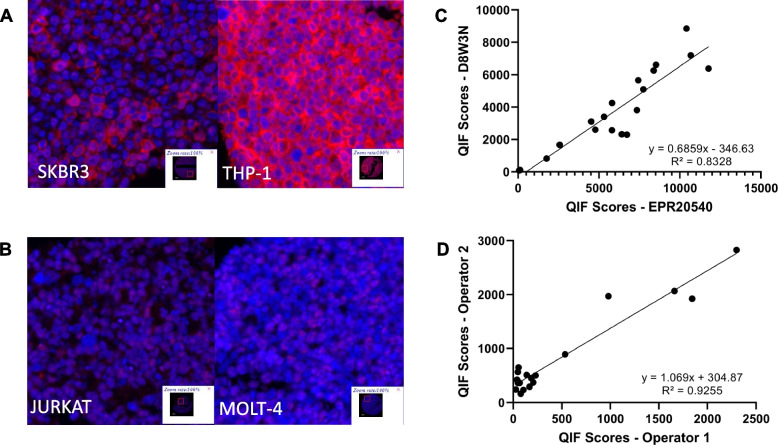
Elements of antibody validation for immunofluorescence including (**A**) representative images showing CD40 expression in positive cell lines SKBR3 and THP-1 (DAPI in blue, CD40 in red), (**B**) representative images showing lack of CD40 expression in negative cell lines JURKAT and MOLT-4, (**C**) regression between EPR20540 and a different CD40 antibody clone, D8W3N, and (**D**) regression of EPR20540 staining between 2 operators

The antibody used for CD40, clone EPR20540, was rigorously validated according to the antibody validation protocol for immunofluorescence previously published by our lab [12]. Briefly, serial cuts of a TMA containing CD40-positive and CD40-negative cell lines were stained by immunofluorescence with a range of concentrations spanning 3 logs, and then analyzed using the AQUA™ method of QIF as previously described. The optimal concentration of clone EPR20540 was determined to be 0.085 µg/mL (1:5000 dilution), which was the concentration with both a high signal to noise ratio (determined by dividing the average QIF scores of the highest 10% of spots by the average QIF scores of the lowest 10% of spots) and a strong enough signal to ensure detection across the entire dynamic range. Signal localization to the cell membrane confirmed correct subcellular localization of the antibody, and two positive cell lines (THP-1 and SKBR3) and two negative cell lines (MOLT-4 and JURKAT) provided genetic validation of the antibody (Fig. 1A-B). Allowing the assumption that different antibody clones bind to separate, non-overlapping epitopes, the correlation (R^2^ = 0.83) between clone EPR20540 and clone D8W3N (Cell Signaling Technology, Danvers, MA, USA) further validated the antibody, and the confirmation of reproducibility between operators (R^2^ = 0.93) completed the antibody validation process for CD40 clone EPR20540 (Fig. 1C-D).

### mRNA CD40 expression from The Cancer Genome Atlas (TCGA)

A publicly available TCGA dataset quantifying CD40 mRNA expression by RNA-seq in 17 various cancer types was downloaded from The Human Protein Atlas (https://www.proteinatlas.org/about/download), where it is specified that this dataset is based on The Human Protein Atlas version 22.0 and Ensembl version 103.38. From this dataset, FPKM values were retrieved and used to compare CD40 mRNA expression levels between the 9 cancer types assessed in this study; bladder (TCGA-BLCA; *n* = 406), breast (TCGA-BRCA; *n* = 1075), colon (TCGA-COAD; *n* = 597), gastric (TCGA-STAD; *n* = 354), head and neck (TCGA-HNSC; *n* = 499), NSCLC (TCGA-LUSC; *n* = 994), ovarian (TCGA-OV; *n* = 373), pancreatic (TCGA-PAAD; *n* = 176) and renal (TCGA-KIRC; *n* = 877).

### Statistical analysis

The linear association between two continuous variables was assessed with the Pearson correlation coefficient. A t-test or one-way analysis of variance was used to compare the means between two or more groups, and a chi-square test was used to compare proportions. Statistical significance of the association of CD40 expression with overall survival (OS) was determined using the log-rank test, with OS defined as the length of time from the date of diagnosis to the patient’s decease or the last recorded follow-up. For statistical analysis of OS and clinicopathological characteristics, the data were split into high CD40 expressing and low CD40 expressing groups by median QIF score. Statistical analysis was performed using GraphPad Prism 9.3.1 software, and a two-sided significance level of 0.05 was used for all analyses (GraphPad Software, San Diego, CA, USA).

## Results

### Multi-tumor screen to determine localization and tumor types/histology for CD40 expression

**Fig. 2:**
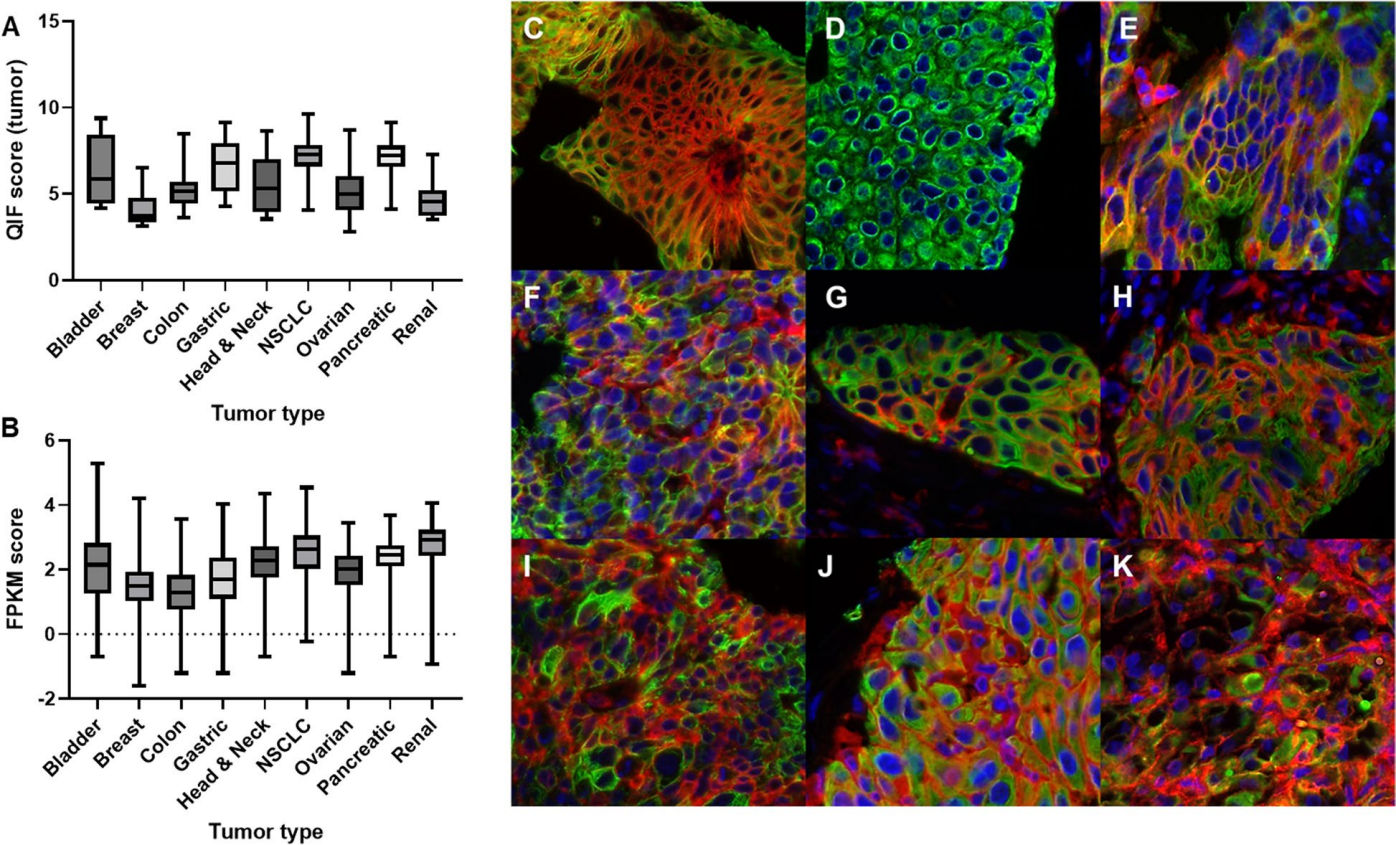
Results from the multi-tumor survey. **A** Natural log-transformed QIF scores representing CD40 protein expression in the tumor compartment across 9 tumor types, (**B**) Natural log-transformed FPKM scores representing CD40 mRNA expression in the whole sample across 9 tumor types, from data publicly available on TCGA, and representative pseudocolor images of CD40 (red) immunofluorescent staining with cytokeratin (green) and DAPI (blue) from (**C**) bladder cancer (**D**) breast cancer (**E**) colon cancer (**F**) gastric cancer (**G**) head and neck cancer (**H**) NSCLC (**I**) ovarian cancer (**J**) pancreatic adenocarcinoma (**K**) renal cancer. (QIF, quantitative immunofluorescence; FPKM, fragments per kilobase of transcript per million mapped reads)

Following rigorous validation, the CD40 antibody clone EPR20540 was used to screen a variety of solid tumor types for CD40 expression on the tumor. As demonstrated by the quantitative expression levels (QIF scores), some degree of CD40 expression in the tumor compartment was found in all the tested tumor types (bladder, colon, gastric, head and neck, NSCLC, ovarian, pancreatic and renal), with the exception of breast cancer (Fig. 2A). Publicly available TCGA data on *CD40* mRNA expression were also analyzed in the same tumor types that were assessed for protein expression by QIF (Fig. 2B). While there was definite variation between the protein and mRNA levels in each cancer type, it is worth noting that while protein expression was measured in each tissue compartment, TCGA transcriptomics data come from bulk RNA sequencing. As seen in the pseudo-color images, CD40 displays a predominantly membranous pattern of staining, with some cytoplasmic staining, and corresponds closely to the cytokeratin staining when it is expressed on the tumor cells (Fig. 2C-K). Following this multi-tumor screening, the large NSCLC, ovarian cancer, and pancreatic adenocarcinoma cohorts were used to further explore patterns of CD40 expression, as well as potential prognostic value and possible correlations with clinicopathologic features.

### Large cohort, population-based assessment of CD40 in NSCLC

**Fig. 3:**
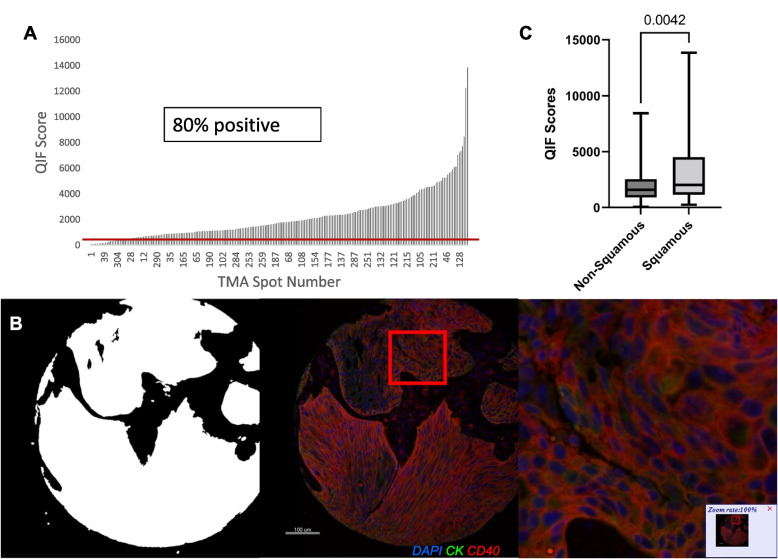
Pattern of expression and distribution of CD40 in NSCLC. **A** Dynamic range of CD40 expression in tumor compartment measured by QIF score with a red line denoting the visual cutpoint, (**B**) representative images showing tumor mask generated from cytokeratin staining, a pseudocolor (nuclei in blue, cytokeratin in green, and CD40 in red) image of a CD40-positive NSCLC case, and 100% zoom on the same pseudocolor image, and (**C**) a comparison of QIF scores between the squamous and non-squamous histological types

Next, CD40 expression on tumor cells was assessed in the NSCLC cohort (YTMA 423). CD40 expression on the tumor membrane displayed noticeable variation between cases, as can be seen from the dynamic range of continuous QIF scores (Fig. 3A). The visual cutpoint was defined as the lowest QIF score that still displayed discernable signal above the background noise and is shown as a red line on the dynamic range (Fig. 3A). In this NSCLC cohort, 80% of the cases were positive for CD40 tumor expression according to the visual cutpoint (Fig. 3A). Figure 3B shows a representative image of a CD40 positive case, as well as an image of the mask used to define the tumor compartment for analysis. Intratumoral heterogeneity of CD40 expression was assessed by comparing two blocks from YTMA423, since each block has a different core from the same case, and notable intratumoral heterogeneity was found in NSCLC (R^2^ = 0.56; Supplementary Fig. 1A). Additionally, CD40 QIF scores were highly concordant between serial sections (R^2^ = 0.89), confirming reproducibility, and showing some correlation between expression in the tumor and stroma compartments (R^2^ = 0.36), indicating that tumors with more CD40 expression in the stroma are more likely to also express CD40 on the tumor surface (Supplementary Fig. 1B, C).

When CD40 tumor expression was assessed for association with clinicopathologic features, the only significant finding was that CD40 tumor expression is higher in squamous NSCLC than non-squamous (*p* = 0.0042; Fig. 3C). No other clinicopathologic features were significantly associated, and no prognostic effect was identified for OS (Table 1, Supplementary Fig. 4A).

### Large cohort, population-based assessment of CD40 in ovarian cancer

**Fig. 4:**
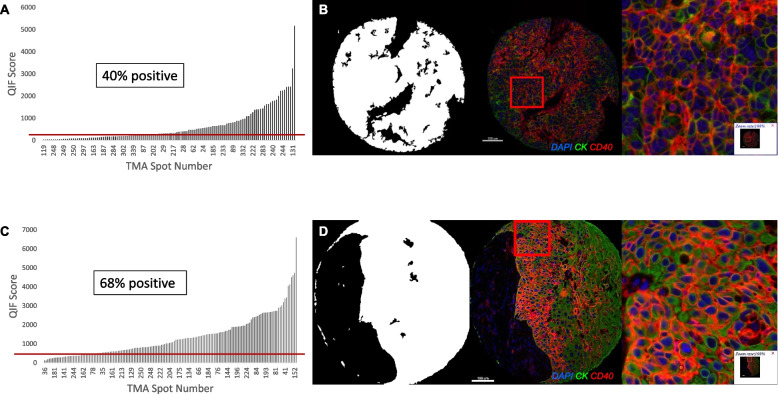
Pattern of expression and distribution of CD40 in ovarian cancer and pancreatic cancer. **A** Dynamic range of CD40 expression in tumor compartment measured by QIF score with a red line denoting the visual cutpoint, and (**B**) representative images showing tumor mask generated from cytokeratin staining, a pseudocolor (nuclei in blue, cytokeratin in green, and CD40 in red) image of a CD40-positive ovarian cancer case, and 100% magnification on the same pseudocolor image. **C** Dynamic range of CD40 expression in tumor compartment measured by QIF score with a red line denoting the visual cutpoint, and (**D**) representative images showing tumor mask generated from cytokeratin staining, a pseudocolor (nuclei in blue, cytokeratin in green, and CD40 in red) image of a CD40-positive pancreatic cancer case, and 100% magnification on the same pseudocolor image

CD40 expression on tumor cells was then similarly assessed in the ovarian cancer cohort (YTMA 264). Similar to that observed in NSCLC, CD40 expression on the tumor membrane displayed noticeable variation between cases, and a wide dynamic range of QIF scores (Fig. 4A). In this ovarian cancer cohort, 40% of the cases were positive for CD40 tumor expression according to the visual cutpoint (Fig. 4A). Figure 4B shows a representative image of a CD40 positive ovarian cancer case. Similar to the NSCLC results, CD40 expression in ovarian cancer showed considerable intratumoral heterogeneity (R^2^ = 0.37), high concordance between serial sections (R^2^ = 0.79), and some correlation between expression in the tumor and stroma compartments (R^2^ = 0.61; Supplementary Fig. 2A-C).

CD40 tumor expression in ovarian cancer was not significantly associated with any of the assessed clinicopathologic features and displayed no significant prognostic effect for OS (Table 1, Supplementary Fig. 4B).

### Large cohort, population-based assessment of CD40 in pancreatic cancer

Finally, CD40 expression on tumor cells was assessed in the pancreatic adenocarcinoma cohort (YTMA 454). Once again, CD40 expression on the tumor membrane displayed noticeable variation between cases, and a wide dynamic range of QIF scores (Fig. 4C). In this pancreatic adenocarcinoma cohort, 68% of the cases were positive for CD40 tumor expression according to the visual cutpoint (Fig. 4C). Figure 4D shows a representative image of a CD40 positive pancreatic adenocarcinoma case, as well as the corresponding tumor mask. In pancreatic adenocarcinoma as well, CD40 expression demonstrated considerable intratumoral heterogeneity (R^2^ = 0.38), high concordance between serial sections (R^2^ = 0.94), and some correlation between expression in the tumor and stroma compartments (R^2^ = 0.30; Supplementary Fig. 3A-C).

CD40 tumor expression in pancreatic adenocarcinoma was not significantly associated with any of the assessed clinicopathologic features and displayed no significant prognostic effect for OS (Table 1, Supplementary Fig. 4C). Since this cohort was comprised only of adenocarcinoma, it was not possible to assess differences in expression between histological types.

## Discussion

Vital in cytotoxic T cell activation and often used as a marker for APCs, CD40 is understandably a molecule of interest in the world of targeted therapies. However, CD40 has been disappointing as a drug target [1, 13]. Considering that CD40-targeting agents are often accompanied by dose-limiting toxicities, this quantitative assessment of CD40 expression by tumor cells may help guide selection of patients and tumor types that would be more likely to benefit from these drugs [6].

CD40 expression has been previously reported in B-cell malignancies, as well as in a variety of solid tumors including bladder cancer, breast cancer, cervical cancer, colon cancer, gastric cancer, head and neck cancer, lung cancer, melanoma, osteosarcoma, ovarian cancer, pancreatic cancer, and renal cell carcinoma [9–11, 14–22]. Within these cancer types, there are conflicting reports on the prognostic significance of tumor cells expressing CD40. For example, CD40 expression on tumor cells was shown to have a negative prognostic effect in NSCLC, while CD40 expression has also been cited as having an anti-tumor effect in several other cancer types, including ovarian cancer [9, 20, 23, 24]. Furthermore, while CD40 expression by tumor cells seems to be common, it has not been assessed quantitatively or in large populations.

After rigorous validation of an antibody for CD40, quantitative immunofluorescence was employed to screen for CD40 expression on tumor cells across nine cancer types, and to complete large cohort, population-based assessments in NSCLC, ovarian cancer, and pancreatic adenocarcinoma. Examples of positive CD40 tumor expression were found in eight out of the nine cancers surveyed, confirming previous reports that this pattern of expression is not limited to only a few cancers [1, 4]. Interestingly, this multi-tumor survey returned a negative result for CD40 expression in breast cancer. In the past, immunohistochemistry-based studies have reported CD40 expression on breast tumors, though using unvalidated, polyclonal CD40 antibodies [14, 25, 26]. The large cohort, population-based assessments demonstrated the variability of CD40 expression by tumor cells within NSCLC (80%), ovarian cancer (40%), and pancreatic adenocarcinoma (68%). A previous assessment of NSCLC tumor cells expressing CD40 had cited a 51.9% (*n* = 129) positivity rate, while a previous pancreatic cancer assessment had reported a 69.2% (*n* = 26) positivity rate [9, 11]. The difference in CD40 expression findings may be the result of the larger population size of this study, or the different methods of assessment (qualitative/semi-quantitative assessment vs. quantitative assessment in molecularly defined tissue compartments). This CD40 tumor expression showed marked heterogeneity between different cores of the same tumor, and partial correlation between the expression of CD40 by tumor cells and the expression of CD40 by stromal cells. The correlation between CD40 expression on tumor cells and on stromal cells was particularly pronounced in ovarian cancer (R^2^ = 0.61), while it was less prominent in NSCLC (R^2^ = 0.36) and pancreatic adenocarcinoma (R^2^ = 0.30). CD40 expression on the tumor epithelium showed no prognostic effect in any of the three large populations assessed. The only significant correlation with clinicopathologic features was the association between the squamous subtype of NSCLC and higher QIF scores for CD40 in the tumor compartment when compared to the non-squamous subtypes (3C).

This study has several limitations. First, all the samples assessed were in the tissue microarray format, which might limit the detection of potential tissue heterogeneity and thus, validation of our findings in whole tissue sections would be useful. Furthermore, the NSCLC cohort used is predominately comprised of early-stage patients with surgically resectable disease, which both limited the assessment of any correlation between CD40 expression and tumor stage and prevented the evaluation of CD40 distribution and CD40 as a prognostic marker in advanced stage disease. Additionally, the pancreatic adenocarcinoma cohort only allowed for assessment of a single histological type, which prevented a comparison of CD40 expression between different subtypes of pancreatic cancer. Finally, this study used a retrospective collection of tissue without standardization of treatment.

## Conclusions

In conclusion, this study demonstrates the quantitative, protein-based assessment of CD40 expression by tumor cells, with a particular focus on NSCLC, ovarian cancer, and pancreatic adenocarcinoma. The detection of CD40 expression in tumor cells needs to be addressed for this targetable molecule to be effectively implemented in cancer therapeutics, with the goal of informing the development of less toxic, yet more potent, future pharmaceuticals and optimizing patient selection in future clinical trials.

## Supplementary Information

**Additional file 1. Supplemental Table 1.** Overview of TMAs used for the multi-tumor survey.

**Additional file 2. Supplementary Figure 1.** Regressions from the NSCLC cohort data showing (**A**) heterogeneity between blocks in tumor, (**B**) reproducibility of QIF scoring in the tumor compartment, and (**C**) the correlation between CD40 expression in tumor versus stroma. **Supplementary Figure 2.** Regressions from the ovarian cancer cohort data showing (**A**) heterogeneity between blocks in tumor, (**B**) reproducibility of QIF scoring in the tumor compartment, and (**C**) the correlation between CD40 expression in tumor versus stroma. **Supplementary Figure 3.** Regressions from the pancreatic cancer cohort data showing (**A**) heterogeneity between blocks in tumor, (**B**) reproducibility of QIF scoring in the tumor compartment, and (**C**) the correlation between CD40 expression in tumor versus stroma. **Supplementary Figure 4.** Kaplan-Meier curves for CD40 expression in (**A**) NSCLC, (**B**) ovarian cancer, and (**C**)pancreatic adenocarcinoma, divided into low CD40 expression and high CD40 expression categories by median.

## Data Availability

All data analyzed/used in the present study are uploaded in Yale AQUAmine repository and also available in the supplementary material of the present manuscript in the form of csv and excel worksheet files.

## Abbreviations

NSCLC: Non-small cell lung cancer
CD40L: CD40 ligand
NK: Natural killer cells
QIF: Quantitative immunofluorescence
TMA: Tissue microarray
DAPI: 4’,6-Diamino-2-phenylindole
OS: Overall survival
