# Supplementary material for: Spatial characterization and quantification of CD40 expression across cancer types
Source: BMC Cancer. 2023 Mar 9;23:220. doi: 10.1186/s12885-023-10650-7 (PMC9996913; doi:10.1186/s12885-023-10650-7)
Supplement: Supplementary file 1 — Additional file 1. Supplemental Table 1. Overview of TMAs used for the multi-tumor survey. [file 12885_2023_10650_MOESM1_ESM.docx]

| **Cohort** | **Number (*N*)** | **Histology** |
| --- | --- | --- |
| YTMA 286 | 24 | Bladder |
| YTMA 417 | 25 | Breast |
| YTMA 260 | 25 | Colon |
| YTMA 142 | 38 | Gastric |
| YTMA 275 | 32 | Head & Neck |
| YTMA 423 | 287 | NSCLC |
| YTMA 264 | 339 | Ovarian |
| YTMA 454 | 238 | Pancreatic Adenocarcinoma |
| YTMA 473 | 21 | Renal |

**Supplemental Table 1**. Overview of TMAs used for the multi-tumor survey.
