# Supplementary material for: Spatial characterization and quantification of CD40 expression across cancer types
Source: BMC Cancer. 2023 Mar 9;23:220. doi: 10.1186/s12885-023-10650-7 (PMC9996913; doi:10.1186/s12885-023-10650-7)
Supplement: Supplementary file 2 — Additional file 2. Supplementary Figure 1. Regressions from the NSCLC cohort data showing (A) heterogeneity between blocks in tumor, (B) reproducibility of QIF scoring in the tumor compartment, and (C) the correlation between CD40 expression in tumor versus stroma. Supplementary Figure 2. Regressions from the ovarian cancer cohort data showing (A) heterogeneity between blocks in tumor, (B) reproducibility of QIF scoring in the tumor compartment, and (C) the correlation between CD40 expression in tumor versus stroma. Supplementary Figure 3. Regressions from the pancreatic cancer cohort data showing (A) heterogeneity between blocks in tumor, (B) reproducibility of QIF scoring in the tumor compartment, and (C) the correlation between CD40 expression in tumor versus stroma. Supplementary Figure 4. Kaplan-Meier curves for CD40 expression in (A) NSCLC, (B) ovarian cancer, and (C)pancreatic adenocarcinoma, divided into low CD40 expression and high CD40 expression categories by median. [file 12885_2023_10650_MOESM2_ESM.pdf]

SF 1

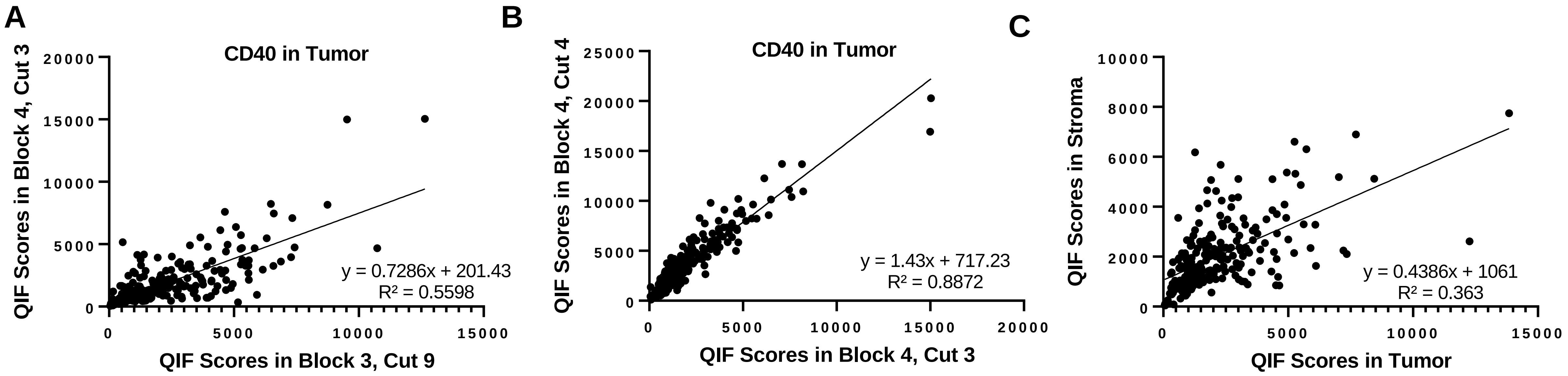

Supplementary Figure 1. Regressions from the NSCLC cohort data showing (A) heterogeneity between blocks in tumor, (B) reproducibility of QIF scoring in the tumor compartment, and (C) the correlation between CD40 expression in tumor versus stroma.

SF 2

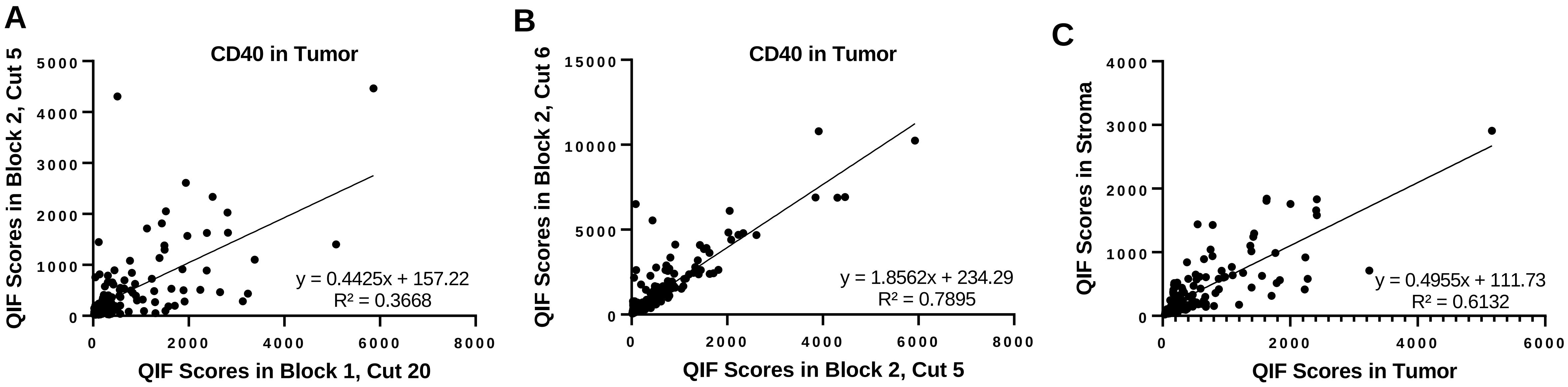

Supplementary Figure 2. Regressions from the ovarian cancer cohort data showing (A) heterogeneity between blocks in tumor, (B) reproducibility of QIF scoring in the tumor compartment, and (C) the correlation between CD40 expression in tumor versus stroma.

SF 3

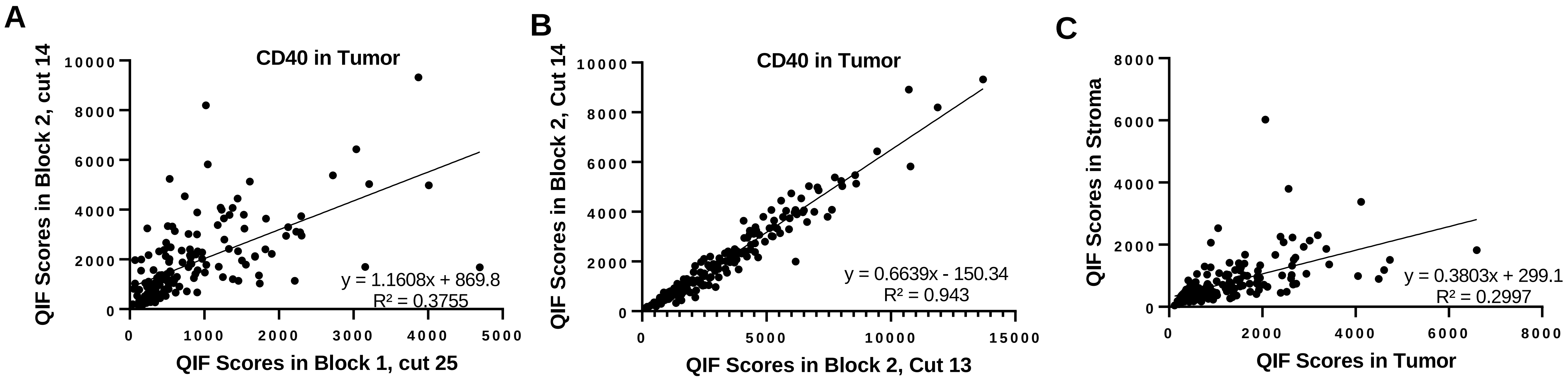

Supplementary Figure 3. Regressions from the pancreatic cancer cohort data showing (A) heterogeneity between blocks in tumor, (B) reproducibility of QIF scoring in the tumor compartment, and (C) the correlation between CD40 expression in tumor versus stroma.

SF 4

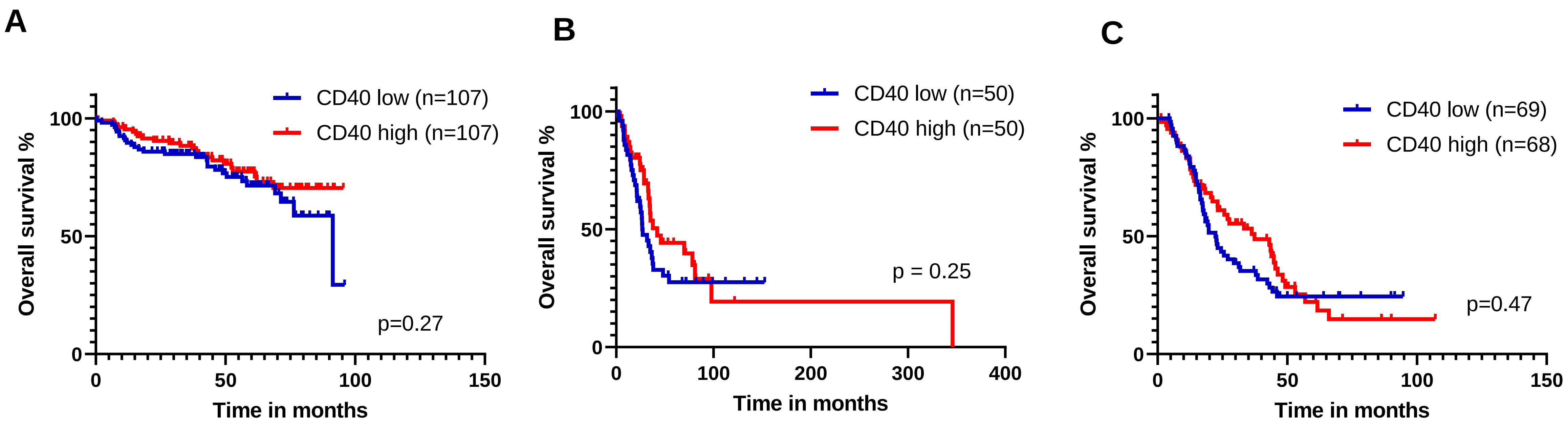

Supplementary Figure 4. Kaplan-Meier curves for CD40 expression in (A) NSCLC, (B) ovarian cancer, and (C) pancreatic adenocarcinoma, divided into low CD40 expression and high CD40 expression categories by median.
